# MicroRNA-598 inhibits the proliferation and invasion of non-small cell lung cancer cells by directly targeting ZEB2

**DOI:** 10.3892/etm.2022.11455

**Published:** 2022-06-20

**Authors:** Xiangdong Tong, Peng Su, Haitao Yang, Fusheng Chi, Lin Shen, Xiao Feng, Hongqian Jiang, Xiuchun Zhang, Zhenyuan Wang

Exp Ther Med 16:5417–5423, 2018; DOI: 10.3892/etm.2018.6825

Following the publication of this paper, it was drawn to the Editors’ attention by a concerned reader that the western blotting data shown in Figs. 3D, 4B and 5A, and several of the panels shown for the cell invasion assays in Figs. 2C and 5C, were strikingly similar to data appearing in different form in other articles by different authors. Owing to the fact that the contentious data in the above article had already been published elsewhere, or were already under consideration for publication, prior to its submission to *Experimental and Therapeutic Medicine,* the Editor has decided that this paper should be retracted from the Journal. The authors were asked for an explanation to account for these concerns, but the Editorial Office did not receive a reply. The Editor apologizes to the readership for any inconvenience caused.

